# Riedel's Thyroiditis: Pitfalls in Diagnosis and Subsequent Complications

**DOI:** 10.1155/2023/9989953

**Published:** 2023-04-15

**Authors:** R. Pandev, M. Khan, V. Ratheesh

**Affiliations:** ^1^Medical University of Pleven, Pleven, Bulgaria; ^2^University Hospital Saint Marina, Pleven, Bulgaria

## Abstract

Riedel's thyroiditis is a rare disease of chronic inflammation with fibrotic infiltration of the thyroid gland and its surrounding vital structures. Due to its low incidence, there are often delays in diagnosis as it is commonly mistaken for other thyroid diseases. We report the case of a 34-year-old female patient who presented with a firm, enlarged mass in the neck, compression symptoms, and hypothyroidism. Lab tests showed elevated A-TG (thyroglobulin antibodies) and A-TPO (thyroid peroxidase antibodies) levels. Based on the disease presentation and supporting lab findings, the patient was misdiagnosed with Hashimoto's thyroiditis and treated accordingly. Yet the patient's symptoms grew progressively worse. She was discovered to have severe tracheal compression and bilateral RLN (recurrent laryngeal nerve) palsy. Tracheotomy became a necessary surgical intervention after the development of respiratory failure, but this procedure was complicated by the development of an intraoperative pneumothorax. After an open biopsy, histology revealed Riedel's thyroiditis. A new treatment was introduced, with which the patient's condition improved. However, she continued to suffer from the open tracheocutaneous fistula left by the tracheostomy, which adversely affected her everyday life. A follow-up operation was performed to close the fistula. In this case report, we discuss the consequences of misdiagnosing the patient and delaying the appropriate treatment for her disease.

## 1. Introduction

Riedel's thyroiditis is a rare inflammatory disease characterized by fibrotic infiltration of the thyroid gland and often its adjacent structures. Over time, the thyroid gland becomes nonfunctional as its parenchyma is replaced by fibrous tissue. The resulting fibrotic thyroid gland is described as nontender with a stony-hard consistency.

As the fibrotic infiltrate progresses, the follicular cells of the thyroid gland are destroyed, commonly leading to hypothyroidism on presentation. Depending on which nearby tissues and structures are affected, the patient may also complain of various obstructive symptoms such as dyspnea or dysphagia.

The diagnosis of Riedel's thyroiditis is a difficult feat. It is a rare disease, and its infrequency often results in its being overlooked as a differential diagnosis. Furthermore, the diagnosis for Riedel's thyroiditis can only be confirmed with an open biopsy of the thyroid gland. So while Riedel's may be suspected based on the patient's clinical presentation, there are no minimally invasive tests or criteria that can be used to establish the diagnosis instead of an open biopsy. This causes delays in diagnosis, which allows for the advancement of fibrotic infiltration into nearby structures. Ultimately, patient discomfort is increased due to otherwise avoidable complications arising from disease progression.

The complications that develop due to the delayed diagnosis and treatment of Riedel's thyroiditis may impede the patient's complete recovery. We report a case of Riedel's thyroiditis where treatment for the patient's disease complications continued long after the patient's Riedel's was successfully managed.

## 2. Case Report

**Figure 1:**
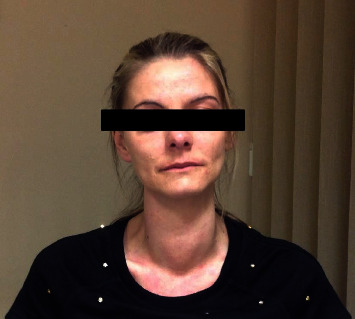
The patient's goiter after her initial visit to the hospital.

A 34-year-old woman presented to the hospital complaining of weight gain, dry skin, hair loss, cold intolerance, extreme fatigue, and a growing neck mass. Upon physical examination of the goiter (Figure 1), it appeared to be very firm to touch. Routine laboratory testing was done, the results of which were typical of a patient with hypothyroidism. TSH > 8 mIU/l, A-TG = 2873 IU/ml, A-TPO = 218 IU/ml, and elevated CRP and ESR levels. On the basis of the clinical picture, a diagnosis of Hashimoto's thyroiditis was made. The patient was treated with L-thyroxine 100–150 mcg daily. This treatment continued for two years without any improvement in the patient's symptoms. The enlargement of the hardened thyroid gland continued. The patient also developed symptoms of compression—dyspnea, dysphagia, and voiding of the voice—that grew progressively worse during this time.

**Figure 2:**
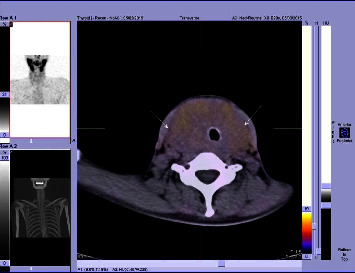
M99Tc SPECT CT scan showing the profusely enlarged goiter with retrotracheal disposition. The trachea is compressed and displaced to the right.

Investigations into the compression symptoms were made. An ultrasound and CT scan were performed. Imaging showed hyperplasia of both lobes of the thyroid gland with marked retrotracheal and retroclavicular disposition (Figure 2). There was also deformation of the larynx, as well as compression and displacement of the trachea. A follow-up laryngoscopy confirmed the bilateral paramedian position of the vocal cords and two-sided laryngeal nerve palsy. Despite the attending doctor's recommendation, a fine needle aspiration biopsy (FNAB) of the thyroid gland was not performed as per the patient's request.

Not long after, the patient was admitted to the hospital with bilateral RLN palsy and clinical symptoms of respiratory failure. The patient was immediately operated on–a joint tracheostomy and an open biopsy of the thyroid were performed. During this operation, a right-sided pneumothorax occurred as an iatrogenic interoperative complication, which was quickly treated with thoracocentesis.

The histological sample from the open biopsy was assessed, and the diagnosis of Riedel's thyroiditis was made. The sample was sent for a second opinion where the diagnosis was reconfirmed.

A treatment plan was devised keeping in mind the new diagnosis of Riedel's thyroiditis. The patient was prescribed L-thyroxine, methylprednisolone, tamoxifen, and mycophenolate mofetil. The patient reacted poorly to the mycophenolate mofetil, but continued treatment with the remaining medication. Over the next 3 years, the patient's goiter significantly reduced in volume. Neck ultrasonography was performed, which showed hypoechoic tissue was present over the glandular isthmus. The rima glottis of the right vocal cord was stationary. Examination confirmed the permanent paralysis of the right RLN. Lab tests were also performed, which showed that CRP and ESR levels had normalized.

Under the new medical regime, the patient's thyroiditis and hypothyroidism were under control. However, the patient was in discomfort from a tracheocutaneous fistula that had developed as a complication of the tracheotomy. A flexible laryngoscopy and tracheoscopy were used to determine that the lumen of the rima glottis was wide enough to close the tracheostomy. The patient's fistula was closed, and the postoperational checkup was favorable.

**Figure 3:**
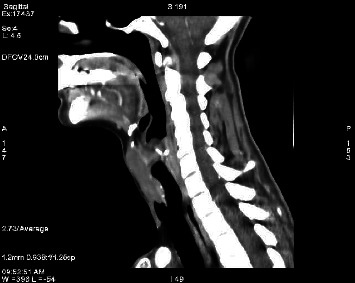
CT scan 6 months post-op showing successful reduction in goiter size through medical treatment.

A follow-up evaluation at the 6-month mark showed no tracheal narrowing on the CT scan (Figure 3). Since then, the patient has had a normal quality of life without any signs of recurrence of symptoms at her annual check-ups. She continues to take L-thyroxine 125 mcg daily for her hypothyroidism, but does not require any other medication. At this moment, the patient is euthyroid with normal TSH levels.

## 3. Discussion

Riedel's thyroiditis is a very rare condition and therefore difficult to diagnose. It has a very low incidence, estimated at 1.06 cases per 100,000 people [1, 2]. Because of its scarcity, it is easy to consider other, more common thyroid disorders before Riedel's thyroiditis. In literature, Riedel's thyroiditis has only ever been described in case reports or case series [3], and no pathognomonic presentation has been established [4, 5]. Case reports show that patients present with a variety of signs and symptoms such as a goiter [1, 2, 6], pain, dysphagia, vocal cord paralysis, tracheal narrowing, and extracervical fibrosis [3]. Some of the signs and symptoms of Riedel's thyroiditis overlap with those of other thyroid disorders [7]. In particular, the clinical presentation of Riedel's thyroiditis is often said to mimic malignancy [8], mainly anaplastic carcinoma [9, 10]. Misdiagnosis with closely resembling diseases can also contribute to the impediment of reaching the correct diagnosis. On average, the diagnosis from initial symptom presentation to histological confirmation can range from 10 months to 2 years [2]. As a result of delayed diagnosis, compression symptoms such as stridor, Horner's syndrome, and venous sinus thrombosis can also develop [1, 2] from hyperplasia of the gland and fibrotic infiltration into nearby structures.

In this case report, the patient's goiter had a stony consistency, similar to how the thyroid was originally described in this disease as “eisenharte” (iron-hard) [2]. Hypothyroidism develops in 25–80% of patients over the course of their disease [2] as the thyroid gland becomes gradually fibrosed [11]. Similar to our patient, 90% of other Riedel's thyroiditis patients present with elevated TG and TPO levels [1, 2, 7], as well as with elevated inflammatory markers CRP and ESR [6]. Riedel's thyroiditis has also been found to occur more commonly in smokers than in nonsmokers [3], and since our patient had a 15 year history of being a heavy smoker, this increased her risk of developing Riedel's. The initial misdiagnosis of Hashimoto's thyroiditis was made based on the patient's clinical presentation and lab results, and treatment with L-thyroxine was started.

The progression of the disease under this incorrect treatment eventually led to acute respiratory failure due to compression of the trachea from the growing goiter. Due to the life-threatening nature of acute respiratory failure, an emergent tracheotomy was performed. However, the haste in performing this procedure led to the iatrogenic intraoperative complication of pneumothorax. The patient's pneumothorax was quickly corrected with thoracocentesis. Once the patient's condition stabilized, an open biopsy was performed, through which the diagnosis of Riedel's thyroiditis was made. An open biopsy is the only way to confirm the diagnosis of Riedel's thyroiditis [1, 2]. Histology from the biopsy showed thyroid parenchyma replaced by dense, fibrous tissue containing inflammatory cells such as eosinophils, lymphocytes, and plasma cells which is typical for Riedel's thyroiditis [1, 2, 6]. Inflammatory cells are commonly seen in Riedel's thyroiditis histology, but giant and malignant cells should not be present [1, 2]. The histological sample was sent to two different laboratories to corroborate the diagnosis.

After the diagnosis, the patient was started on corticosteroid therapy: methylprednisolone 24 mg. Although there is no consensus for the management of Riedel's thyroiditis due to the rarity of the disease, corticosteroids are often used as first-line treatment. Glucocorticoids often result in a dramatic improvement of symptoms, particularly if they are initiated early in the course of the disease [1, 2]. The patient was also prescribed tamoxifen (2 × 10 mg). Tamoxifen is often used either in conjugation with or as an alternative to corticosteroid therapy [11]. The mechanism of action of tamoxifen in the treatment of Riedel's thyroiditis is thought to be unrelated to its antiestrogen activity [12], but rather through its production of TGF-*β* which inhibits fibroblastic growth [13]. Tamoxifen is effective in decreasing the goiter mass and compression symptoms of Riedel's [14] and is a safe long-term therapy option [15].

Less commonly, alternative medical treatments have also been used in the treatment of Riedel's thyroiditis. Case reports have mentioned successful treatment with mycophenolate mofetil, rituximab, and low dose radiation [16]. These therapies are often considered in refractory cases. Mycophenolate mofetil is an immunosuppressant that was recommended to our patient because it has antifibrosing properties [1, 2]. However, this drug was quickly discontinued from the patient's line-up because it was poorly tolerated. Mycophenolate mofetil is associated with several serious side effects, including pancytopenia and renal failure [17]. A drug gaining popularity in the treatment of refractory Riedel's thyroiditis is Rituximab, a monoclonal antibody against the CD20 protein found mainly on B lymphocytes. It is the therapy of choice after the failure of conventional therapies [17] and has been successful in several case reports [18, 19]. Low-dose radiation therapy has also been used as a treatment option in cases of total refractory Riedel's thyroiditis, although its efficacy has not been established [5].

Extensive surgery is not considered a viable treatment option because of the thyroid gland's fibrous infiltration into nearby structures, making it nearly impossible to resect [1, 2, 20]. Total thyroidectomies are associated with an increased risk of complications in up to 39% of cases [1], including disease recurrence [21, 22]. Therefore, surgical intervention is limited to debulking and is only indicated to relieve compression symptoms or when alternative therapies have failed [1, 2]. Isthemectomies and subtotal thyroidectomies are usually the procedure of choice [2, 6].

In the case of our patient, only medical therapy was necessary. The patient was treated with corticosteroid, tamoxifen, and L-thyroxine, which were continued over the course of 3 years during which the patient's condition steadily improved. The patient's goiter reduced in size, and the lab values for inflammatory markers fell within the normal range.

Despite the successful management of her Riedel's thyroiditis, the patient continued to suffer. A tracheocutaneous fistula had formed as a complication of the tracheostomy that was done to relieve her compression symptoms. Tracheocutaneous fistulas occur as a complication of tracheostomies at a rate of 1% [23]. This fistula was a source of discomfort for the patient, and it reduced her quality of life. The fistula was evaluated using a flexible laryngoscope and tracheoscope in order to see if surgical closure was possible. Once the rima glottis was found to be wide enough, the patient's fistula was closed without any complications.

The patient was able to completely recover from the operation and now has a normal quality of life. She continues to take L-thyroxine for her hypothyroidism but has discontinued all other medication.

Recurrence of Riedel's thyroiditis following cessation of medication is not uncommon [2, 11], so the patient is evaluated biannually to check for relapse. As of yet, there is no sign of the disease returning.

## 4. Conclusion

Riedel's thyroiditis often has a delayed diagnosis because its clinical presentation may be difficult to differentiate between other, more prevalent thyroid disorders. Delays in diagnosis can lead to uninhibited disease progression, which has higher rates of complications and mortality.

Once Riedel's thyroiditis is diagnosed, the inflammatory process is often treated with high dose glucocorticoids followed by tamoxifen [15], and is said to have a good prognosis [1]. If the patient presents with hypothyroidism, lifelong hormone replacement therapy with L-thyroxine also needs to be initiated. In this case report, our patient was successfully treated with the aforementioned therapies, and did not require the use of alternative medical therapies or surgical intervention.

The surgical operations performed on her, the tracheotomy and the closure of the tracheocutaneous fistula, were a result of complications from a late diagnosis. This case report highlights the importance of including rare diseases such as Riedel's thyroiditis as part of a differential diagnosis. Despite their infrequency, rare diseases must be considered, particularly in patients that are not benefitting from their prevailing treatment plan. On the basis of this case report, we suggest that Riedel's thyroiditis should be considered in the differential diagnosis of patients presenting with stony-hard goiter and compression symptoms. It is important to familiarize ourselves with the clinical presentation of Riedel's thyroiditis so that we can recognize the disease signs and symptoms early. Making an accurate and timely diagnosis will hasten the patient's recovery as well as help decrease the risk of developing late disease complications.

## Data Availability

The data used to support the findings of this study are included within the article.
